# Rescue Therapy for Impacted Large Chicken Bone in the Esophagus: Argon Plasma Coagulation–Assisted Endoscopic Removal – A Case Report

**DOI:** 10.5152/tjg.2025.25481

**Published:** 2025-11-03

**Authors:** Muhammet Yener Akpinar, Hasan Sahin, Tolga Bakir, Mustafa Gulsen

**Affiliations:** Department of Gastroenterology, Ankara Gülhane Training and Research Hospital, Ankara, Türkiye

**Dear Editor,**

A 46-year-old female with no history of systemic or psychosocial issues presented with a complaint of foreign body sensation in her throat after drinking chicken soup. The informed consent was obtained. She denied prior episodes of foreign body ingestion or any relevant family history. She reported pain and a feeling of obstruction while swallowing. Physical examination and laboratory tests were unremarkable except for leukocytosis (14.7 × 10^9^/L) and an elevated C-reactive protein (CRP) level (19 mg/L).

Neck computed tomography (CT) revealed a dense foreign body measuring 21 × 11 mm within the upper esophageal lumen, causing near-complete luminal obstruction. Due to the detection of complete blockage and suspicion of mediastinitis, initial evaluation included consultations with infectious disease, general surgery, and thoracic surgery departments. Empiric antibiotherapy was initiated, and an endoscopic approach was preferred. During the initial endoscopic evaluation, a chicken bone approximately 2 cm in diameter was identified in the proximal esophagus. Following the complete obstruction and the rigid, potentially lacerating nature of the foreign body, attempts at removal using a snare, balloon, and standard forceps were unsuccessful. The patient was closely monitored in the intensive care unit and underwent a second endoscopic procedure under general anesthesia. As part of the rescue therapy, an initial attempt was made to create access using a needle-knife sphincterotome, but this was unsuccessful because of the hardness and position of the foreign body. Subsequently, a second rescue technique was employed using an argon plasma coagulation (APC) probe (KLS MARTIN ME 411) set to 60 watts. Two linear tunnels were created on the surface of the bone, facilitating better instrument engagement. These grooves enabled secure grasping with alligator-jaw forceps, and the foreign body was successfully extracted without complication, as shown in Figure 1.

Follow-up CT revealed no signs of complications other than a localized collection thought to be secondary to trauma, as seen in Figure 2. The case was clinically stable, and she was discharged. At the one-month follow-up endoscopy, no significant pathology was observed, the patient recovered fully, and no long-term complications were expected.

In line with the European Society of Gastrointestinal Endoscopy guidelines,^1^ computed tomography was utilized to assess potential complications, surgical consultation was obtained prior to the innovative endoscopic approach, and urgent endoscopic intervention was preferred as a result of complete obstruction by a sharp foreign body.

Argon plasma coagulation is used in various procedures such as the removal of embedded stents, ablation of granulation tissue around metal stents, control of bleeding after sphincterotomy, debulking of periampullary tumors, coagulation of superficial mucosal lesions, and endoscopic hemostasis.^2^ Similar to previously reported cases involving difficult foreign body extraction, such as the entangled plastic wires described by Park et al,^3^ our case also required the use of multiple endoscopic tools in a non-standard fashion. In Park’s case, APC was used to damage the food material surrounding plastic wires, indirectly weakening the structure. Benatta et al^4^ described the use of APC and polypectomy snare to fragment and remove a gastric trichobezoar in a pediatric patient, avoiding surgery. A recent case by Abadia et al^5^ demonstrated successful removal of an impacted garlic clove in the esophagus using a similar APC-based tunneling technique, followed by Fogarty balloon extraction. Our approach parallels this strategy but differs in target material (bone vs. vegetable) and retrieval method (forceps vs. balloon), underscoring the adaptability of APC-assisted tunneling in managing complete esophageal obstructions. All aforementioned cases highlight the potential of adapting endoscopic techniques beyond their conventional use, especially in complex scenarios where traditional methods fail. To the best of our knowledge, this is the first case describing APC-assisted tunneling for complete esophageal obstruction by a chicken bone. This report is limited by being a single case with a relatively short follow-up period. Also, the delayed intervention may have influenced the outcome.

## Figures and Tables

**Figure 1.**
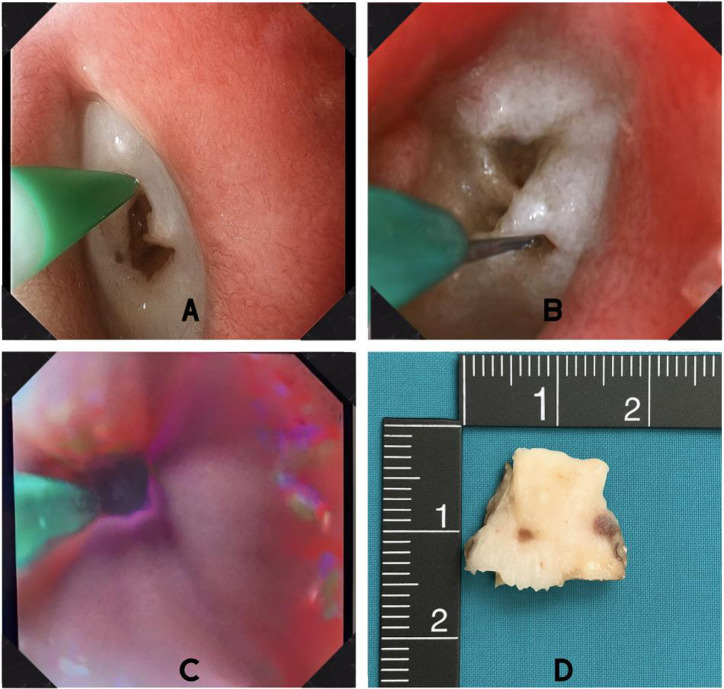
(A and B) Creating a recess in the bone by using an argon plasma coagulation. (C) Formation of a tunnel on the impacted object via argon plasma coagulation. (D) The extracted chicken bone following endoscopic removal.

**Figure 2.**
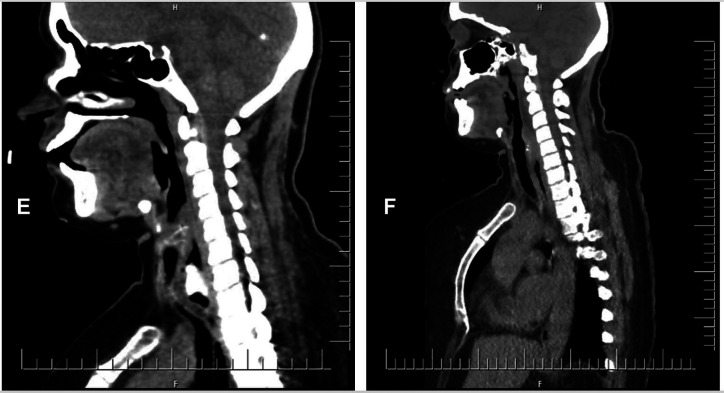
(E) A dense foreign body (chicken bone) impacted in the upper esophagus, causing significant luminal obstruction and surrounding soft tissue edema. (F) P shows post-endoscopic resolution of the obstruction with restored esophageal lumen patency and no signs of perforation or mediastinal complications.

## Data Availability

The data supporting the findings of this case report, including imaging and procedural records, are available from the corresponding author upon reasonable request.
